# OCT Biometry (B-OCT): A New Method for Measuring Ocular Axial Dimensions

**DOI:** 10.1155/2019/9192456

**Published:** 2019-08-14

**Authors:** Bartosz L. Sikorski, Pawel Suchon

**Affiliations:** ^1^Department of Ophthalmology, Nicolaus Copernicus University, 9 M. Sklodowskiej-Curie St., Bydgoszcz 85-309, Poland; ^2^Oculomedica Eye Center, 9 Broniewskiego St., Bydgoszcz 85-316, Poland

## Abstract

**Purpose:**

To present a new method of measuring ocular axial dimensions, termed OCT biometry (B-OCT).

**Design:**

Observational cross-sectional study and evaluation of new diagnostic technology.

**Methods:**

B-OCT was implemented in the spectral domain OCT device for posterior and anterior segment imaging (REVO NX, Optopol Technology). A total of 349 eyes (214 of healthy subjects, 115 of patients with cataract, and 20 with severe macular diseases) were enrolled in the study. The results of B-OCT were compared to swept source OCT-based IOLMaster 700 (Carl Zeiss Meditec). Differences in measurement values between the two biometers were determined using the paired *t*-test. Agreement was assessed through intraclass correlation coefﬁcients (ICCs) and Bland–Altman plots.

**Results:**

B-OCT obtained with REVO NX provides excellent interobserver reproducibility (ICC for: axial length (AXL) = 1.000; central corneal thickness (CCT) = 0.933; anterior chamber depth (ACD) = 0.933; lens thickness (LT) = 0.985) and intraobserver repeatability (ICC for: AXL = 1.000; CCT ≥ 0.994; ACD = 0.998; LT ≥ 0.993). The correlation between measurements made using both devices was outstanding (ICC for: AXL, healthy = 1.000; AXL, cataractous = 1.000; ACD, healthy = 0.998; ACD, cataractous = 0.997; LT, healthy = 0.998; LT, cataractous = 0.997; CCT, healthy = 0.989; CCT, cataractous = 0.979). The mean AXL measurement difference in healthy eyes was −0.001 ± 0.016 mm (the 95% LoA ranged from −0.034 to 0.031); mean ACD difference was 0.000 ± 0.024 mm (95% LoA, −0.047 to 0.047); mean LT difference was −0.002 ± 0.024 mm (95% LoA, −0.050 to 0.046); and mean CCT difference  was −0.8 ± 5.1 *μ*m (95% LoA, −10.81 to 9.26). The mean AXL measurement difference in cataractous eyes was −0.003 ± 0.022 mm (95% LoA, −0.046 to 0.039); mean ACD difference was 0.003 ± 0.029 mm (95% LoA, −0.054 to 0.059); mean LT difference was −0.002 ± 0.025 (95% LoA, −0.051 to 0.048); and mean CCT difference was 2.7 ± 6.4 *μ*m (95% LoA, −9.80 to 15.7).

**Conclusion:**

The study shows small, nonsignificant differences between the biometric measurements obtained with REVO NX B-OCT and IOLMaster 700, which is of high significance for IOL power selection. As B-OCT utilizes a conventional OCT device, the measurements of the ocular axial dimensions are combined with high-resolution macular scans for the simultaneous assessment of central retina as a part of screening for macular pathology. The presented method is the first spectral domain OCT-based biometry technique and the only one integrated into a standard OCT device. Thus, it brings novel functionality to OCT technology.

## 1. Introduction

Accurate measurement of the axial dimensions of the eye (i.e., ocular biometry), and in particular its length, is one of the key parameters used in intraocular lens (IOL) power calculation [1–3]. It also enables the assessment of spatial relationships between ocular structures along the visual axis. Currently, ocular biometry utilizes ultrasound methods with and without immersion, as well as optical techniques. Whereas the former generally offer a better penetration through dense optical media, the latter are noncontact and more precise. Since the launch of IOLMaster (Carl Zeiss Meditec AG, Germany) in 1999, which was based on partial coherent interferometry (PCI), optical biometry has become a gold standard in ocular axial length measurement [4]. Subsequently, other optical biometers became available, such as Lenstar (Haag-Streit Diagnostics, Switzerland) [5], AL-Scan (Nidek Co., Ltd., Japan) [6], Galilei G6 (Ziemer Ophthalmic Systems AG, Switzerland), OA-1000 (Tomey Corp., Japan) [7], and Aladdin (Topcon Corp., Japan) [8, 9]. These devices used either PCI technology or optical low-coherence reflectometry (OLCR). Recently, the optical measurement of ocular axial length has gained an innovative, new generation IOLMaster device (IOLMaster 700, Carl Zeiss Meditec AG, Germany,/*λ* = 1050 nm/), which is the first swept source (SS) optical coherence tomography- (OCT-) based biometer. It enables OCT imaging across the entire length of the eye allowing the operator to view a complete longitudinal cross section through the eye. However, it was developed as an optical biometer and as such does not have the functionalities of conventional OCT devices. On the contrary, none of the devices intended for posterior segment imaging, whether SS- or spectral domain-based, offers biometric measurements. Implementing such functionality in commercially available OCT devices would expand their applicability.

The aim of this paper is to present a new, universal method for axial length measurement, referred to as OCT biometry (B-OCT). It was implemented in an existing and commercially available spectral domain OCT device for posterior and anterior segment imaging (REVO NX, Optopol Technology, Poland) but could also be potentially used in other OCT devices in future. To the best of our knowledge, ours is the first ocular biometry method utilizing spectral domain OCT. As a part of the study, we determined the efficacy, precision, reliability, and clinical utility of B-OCT and compared the agreement between values for axial length (AXL), anterior chamber depth (ACD), lens thickness (LT), and central cornea thickness (CCT) obtained using B-OCT (REVO NX) and IOLMaster 700. Measurement failure rates with both devices were also recorded and compared.

## 2. Materials and Methods

### 2.1. Subjects

This prospective study comprised eyes of healthy subjects and eyes of patients with cataract. A total of 349 eyes examined from November 2017 to May 2018 were enrolled. All of them underwent a comprehensive ocular assessment, including subjective refraction, noncontact tonometry, and slit-lamp and fundus examination. In addition, cataract types were recorded as nuclear, cortical, or posterior subcapsular according to Lens Opacities Classiﬁcation III scoring system (LOCS III) [10]. The healthy group consisted of eyes without optic media opacities or other pathology, especially macular diseases. Fifty healthy eyes (mean age 35.4 ± 4.7 years; 26 women, 24 men) were examined to validate the repeatability and reproducibility of B-OCT. Automated B-OCT (REVO NX; *λ* = 840 nm) measurements of the remaining 164 healthy eyes (mean age 40.1 ± 16.3 years; 101 women, 63 men) and 115 cataractous eyes (mean age 68.9 ± 10.5 years; 71 women, 44 men) were compared to biometry values obtained using SS-OCT IOLMaster 700 (*λ* = 1050 nm). If a measurement was not feasible after two attempts, a measurement failure was recorded for a given device. In addition, 20 patients with serious macular abnormalities were examined, which could hinder the assessment of the posterior retinal boundary. The study protocol was in accordance with the Declaration of Helsinki, and the Institutional Ethics Committee approval was obtained.

### 2.2. OCT Biometry

B-OCT enables measurement of ocular axial dimensions using a conventional OCT system. During the examination, the scanning light beam passes through ocular structures located along the visual axis and the following are identified: anterior and posterior boundary of the cornea, anterior and posterior boundary of the lens, as well as the posterior boundary of the retina. As the commercially available OCT devices for posterior and anterior segment imaging do not enable visualising the entire ocular axial structure on a single scan, the proposed biometry approach relies on a precise identification of measured structures which are acquired individually. The measurements are performed in four measurement windows. Each of them is 3 mm wide and 2.5 mm deep and covers a different ocular structure. The first window contains the anterior and posterior boundary of the cornea, the second one the anterior boundary of the lens, the third one the posterior boundary of the lens, and the fourth one the retina. As the anteroposterior dimension of crystalline lens is larger than a single measurement window (2.5 mm), the boundaries of the lens are identified in two windows (the second and the third one). Subsequent cross sections through ocular tissue in consecutive measurement windows are acquired as the imaging window is shifted along the *Z* axis by means of the C-gate shift along the measurement axis.

**Figure 1:**
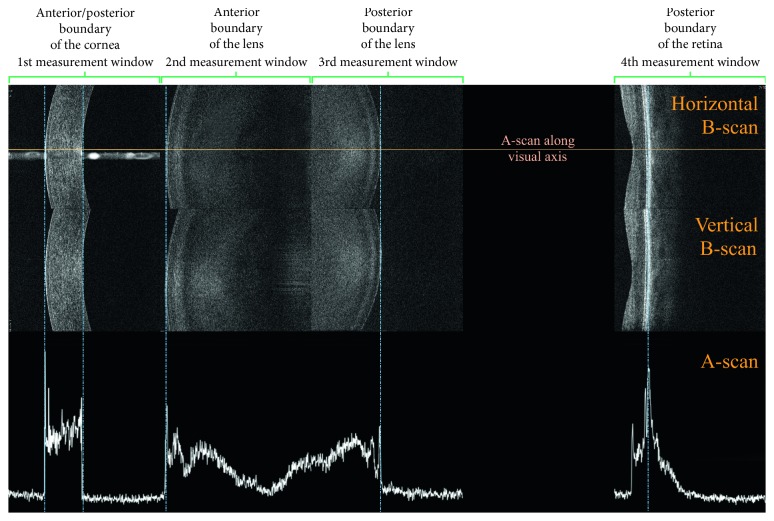
B-OCT measures ocular axial dimensions in four 3 mm wide and 2.5 mm deep measurement windows. In the upper and central parts of the figure, a single horizontal and vertical scan of ocular structures in their respective measurement windows is shown. The lower row presents the corresponding A-scan. The blue vertical lines indicate automatically identified boundaries.

In order to determine the axial length of individual ocular structures, a series of 10 vertical and horizontal measurements are taken. The outliers are rejected by the device, and the mean is computed. The measurement and boundary identification are fully automated. However, all boundaries can also be manually corrected by the clinician if necessary. Should that be the case, the parameter values are recalculated. The clinician can view all measurements with visual presentation of measurement windows and their respective structures as horizontal and vertical B-scans as well as corresponding A-scans (Figure 1).

### 2.3. Instruments

B-OCT was implemented in a commercially available OCT device, REVO NX, by modifying its software and ocular scanning method. In order to determine the accuracy of the device, a series of measurements were carried out using REVO NX and DEA Global Advantage Measurement Machine (Hexagon Manufacturing Intelligence, UK) while assessing the dimensions of the N-BK7 glass block stored for 12 hours at 69.8°F. The measurement values obtained using REVO NX were divided by the averaged index of refraction of the human eye and subsequently multiplied by the refraction index of the N-BK7 glass for the wavelength of REVO NX. The mean thickness of a glass block measured using REVO NX was 29.9964 mm (SD = 0.00168 mm), and the mean thickness obtained using DEA Global Advantage Measurement Machine was 29.9940 mm (SD = 0.000312 mm). After mechanical accuracy of the device was deemed satisfactory, 50 healthy eyes were examined in order to determine intraobserver repeatability and interobserver reproducibility of B-OCT using REVO NX. Each nonmydriatic measurement was taken three times by 2 clinicians at random order, under ambient lighting conditions. Each measurement consisted of 10 shots, and the mean value and SD were displayed automatically at the end of the measurement for AXL, ACD, LT, and CCT. Following each measurement, the subject head was repositioned on the chin rest and the REVO NX device was realigned. The measurements for comparative studies were taken in the same fashion.

The axial length measurements with IOLMaster 700 were the average values of three scans in each of six meridians. The scans were checked for foveal position to ensure the correct axis measurements, and the examination was repeated if the patient was not ﬁxating correctly. The quality control criteria for IOLMaster 700 were applied in accordance with the manufacturer's recommendations.

### 2.4. Statistical Analysis

Statistical analysis was performed using Statistica 13.1 (Dell Inc., USA). To determine the intraobserver repeatability and interobserver reproducibility of the REVO NX B-OCT, the within-subject standard deviation (Sw), test-retest repeatability (TRT), coefficient of variation (CoV), and intraclass correlation coefficient (ICC) were calculated and analysed. The Sw was the square root of the residual mean square in the one-way analysis of variance. The TRT was deﬁned as 2.77 Sw, which shows the interval within which 95% of the differences between measurements are expected to lie [11]. The percentage of CoV was calculated as the ratio of the Sw to the mean. The ICC represented the consistency in data measurement; the high agreement is indicated by a value higher than 0.9 [12]. To assess agreement between REVO NX B-OCT and IOLMaster 700 biometry for AXL, ACD, LT, and CCT, paired samples *t*-test and Bland–Altman plots were performed and 95% limits of agreement were calculated by the mean difference ± 1.96 SD [13]. A *p* value less than 0.05 was considered statistically significant.

## 3. Results

The results of intraobserver repeatability and interobserver reproducibility assessment of B-OCT using REVO NX in 50 healthy eyes are shown in Tables 1 and 2. The ICC of repeatability for AXL was 1.000. For other parameters, i.e., CCT, ACD, and LT, the ICC was also very high (≥0.994, =0.998, and ≥0.993). The ICC of reproducibility for AXL was 1.000 and that for CCT, ACD, and LT was 0.933, 0.933, and 0.985, respectively.

Minimum and maximum values of ocular axial dimensions measured using B-OCT in healthy eyes were for AXL = 19.11–34.48 mm, ACD = 1.65–4.32 mm, LT = 3.30–5.34 mm, and CCT = 0.478–0.682 mm. The same values in cataractous eyes were AXL = 19.62–32.07 mm, ACD = 2.16–3.98 mm, LT = 3.94–5.48 mm, and CCT = 0.438–0.631 mm. In 3 eyes, with the weak signal from the retina due to the dense cataract, the AXL measurements were corrected manually.

**Figure 2:**
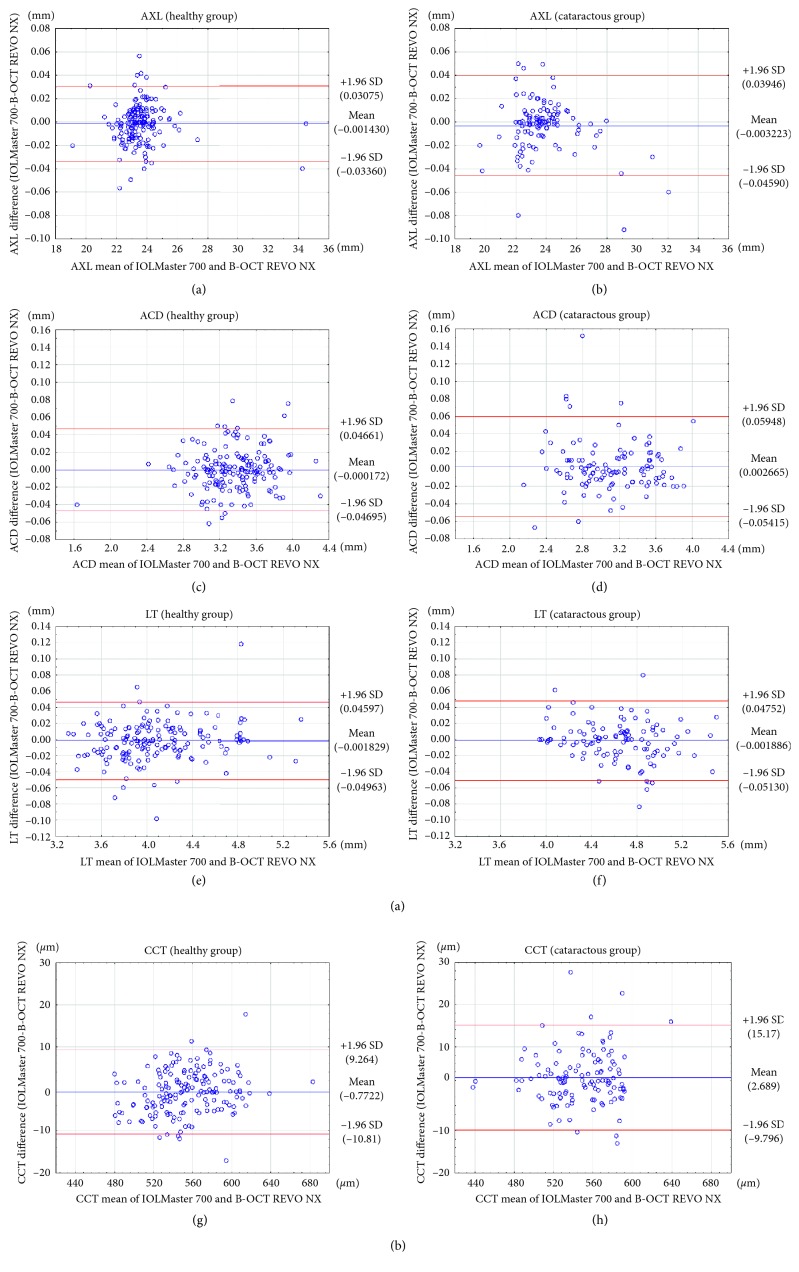
Bland–Altman plots for AXL (a, b), ACD (c, d), LT (e, f), and CCT (g, h). The red lines indicate the 95% agreement interval.

In healthy volunteers, both REVO NX and IOLMaster 700 were able to successfully measure AXL, ACD, LT, and CCT in all eyes within high-quality SD limits of the manufacturers. In cataract patients, IOLMaster 700 had a lower measurement failure rate. AXL measurements were not possible for 4 cataractous eyes with REVO NX, whereas with IOLMaster 700 only in 1 eye. The failure rates when measuring ACD and LT were 3 and 4 vs. 2 and 2 for REVO NX and IOLMaster 700, respectively. Bland–Altman plots for comparisons between IOLMaster 700 and B-OCT obtained with REVO NX values for AXL, ACD, LT, and CCT are presented in Figure 2.  Table 3 shows a comparison of values measured using REVO NX and IOLMaster 700. The mean AXL measurement difference in healthy eyes was −0.001 ± 0.016 mm (the 95% limits of agreement (LoA) on Bland–Altman plots ranged from −0.034 to 0.031); mean ACD difference was 0.000 ± 0.024 mm (95% LoA, −0.047 to 0.047); mean LT difference was −0.002 ± 0.024 mm (95% LoA, −0.050 to 0.046); and mean CCT difference was −0.8 ± 5.1 *μ*m (95% LoA, −10.81 to 9.26). The mean AXL measurement difference in cataractous eyes was −0.003 ± 0.022 mm (95% LoA, −0.046 to 0.039); mean ACD difference was 0.003 ± 0.029 mm (95% LoA, −0.054 to 0.059); mean LT difference was −0.002 ± 0.025 (the 95% LoA, −0.051 to 0.048); and mean CCT difference was 2.7 ± 6.4 *μ*m (95% LoA, −9.80 to 15.7).

**Figure 3:**
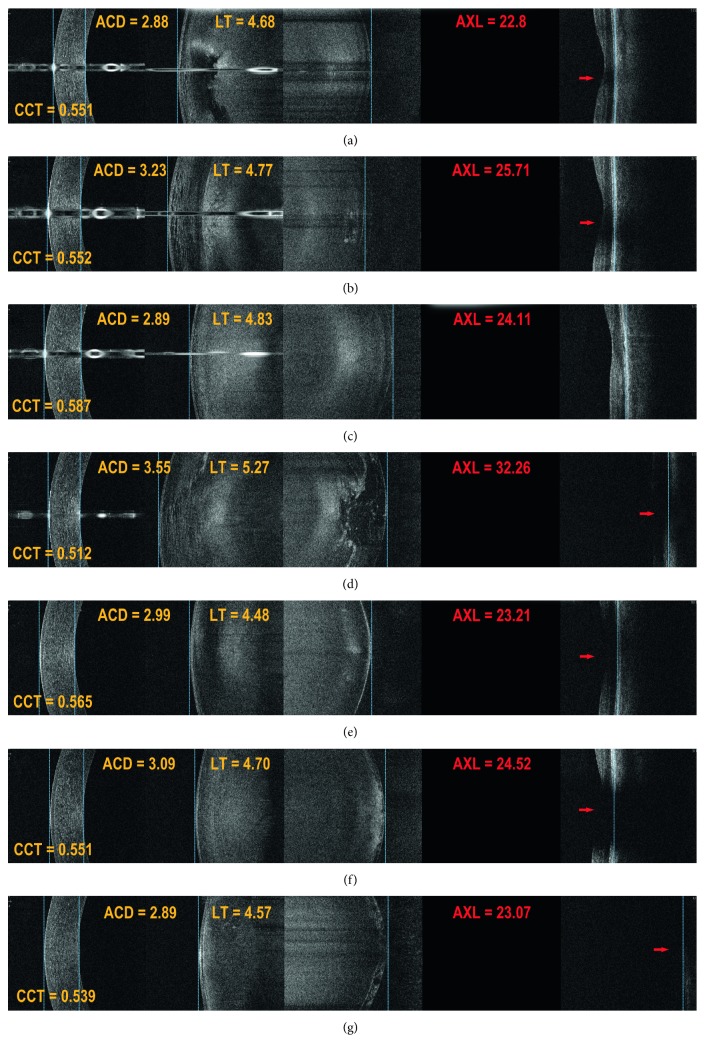
Lens opacity location in B-OCT (REVO NX) and its effect on the visibility of the posterior retinal boundary. (a, b) Opacity of the anterior part of the lens and nucleus; (c) increased lens reflexivity corresponding to nuclear cataract; (d) nuclear and posterior subcortical opacity. Despite significant retinal shading, the posterior retinal boundary remains visible and is accurately identified; (e–g) posterior subcapsular opacity of increasing density degree. A thick subcapsular opacity in (g) cast a major shade on the sensory retina. However, the posterior boundary of the retina was imaged. The red arrows indicate retinal shading due to lens opacity.

**Figure 4:**
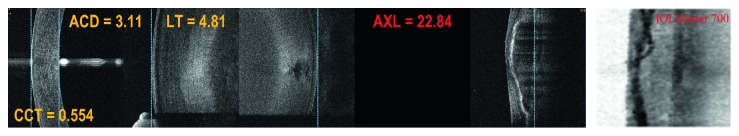
Comparison of ocular structure boundary identification using B-OCT (REVO NX) and IOLMaster 700. Measurement failure of ocular axial length disregarding RPE elevation in a patient with drusenoid RPE detachment using IOLMaster 700. Owing to the possibility of manual RPE marker adjustment, B-OCT is capable of identifying both posterior RPE boundary and original location of elevated RPE.

**Figure 5:**
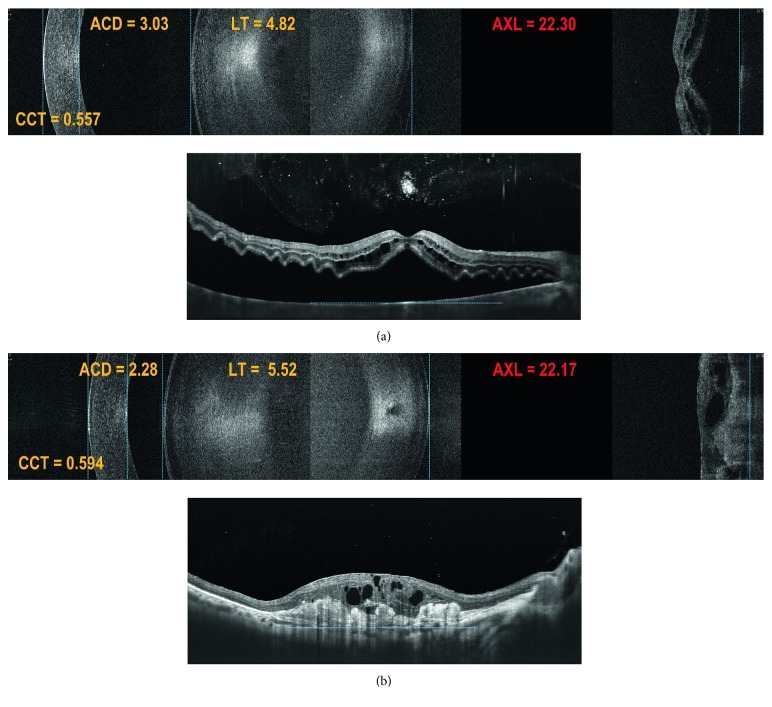
Ocular axial length measured using B-OCT in patients with macular diseases. (a) Correct, precise identification of the posterior boundary of RPE in a patient with retinal detachment. (b) Manual identification of original boundary of RPE in a patient with a disciform scar. The blue lines indicate boundary location.

Figure 3 presents examples of B-scans with lens opacity of various degrees and its effect on the possibility to visualise the posterior boundary of the retina necessary for the AXL measurement. In 17 of 20 challenging macular cases, it was possible to reliably determine the AXL when using manual setup in B-OCT (Figures 4 and 5), whereas with IOLMaster 700, the exact position of the measured retinal boundary was unknown (Figure 4). Three eyes that were not measured successfully with B-OCT had bullous retinal detachment.

## 4. Discussion

Satisfactory refractive outcomes after IOL implantation depend on optimum biometry measurement. The AXL readings are crucial for all IOL power calculation formulas to ensure accurate power estimation. Poor fixation, macular abnormalities, dense lens, and vitreous opacities may all contribute to measurement errors when determining the ocular axial length. SS-OCT IOLMaster 700 partly overcomes these limitations. It provides an image-based measurement, allowing the operator to view the complete longitudinal section of the eye. It also helps identify irregular eye geometries and foveal problems that may alert the operator to insufﬁcient ﬁxation during measurements [14]. That is why, as a new gold standard, the SS-OCT device was chosen as a comparator to B-OCT obtained using REVO NX. The B-OCT had an outstanding correlation with SS-OCT IOLMaster 700 (ICC of 1.000) for AXL measurement both in healthy volunteers and cataract patients. The mean AXL measurement difference in 164 healthy eyes was −0.001 mm (SD = 0.016), as compared to −0.003 mm (SD = 0.022) in 111 cataract eyes. These results show that both devices measure AXL in an almost identical way. The difference is smaller than the one between IOLMaster 700 and IOLMaster 500 [14]. The ICC for ACD and LT measured in both healthy and cataractous eyes was 0.998 and 0.997, respectively. The mean and SD of measurement differences in ACD and LT between the devices were also outstanding in both groups. The difference in ACD values obtained using the two study devices is again smaller than the one between IOLMaster 700 and IOLMaster 500 [14]. Slightly larger difference of the means for CCT may be attributable to pixel size difference, with the pixel size in REVO NX being several-fold smaller than that in IOLMaster 700. It should be noted that IOLMaster 700 enables tissue scanning with 22 *μ*m axial resolution, whereas REVO NX offers the resolution of 5 *μ*m, which is over four times better. Thus, it identifies boundaries of individual structures with higher accuracy, so the value of CCT measured using REVO NX is more stable. However, the higher nominal resolution of REVO NX resulted in no significant difference in the precision of the biometric measurement of REVO NX compared to IOLMaster 700. Considering almost identical standard deviations of repeatability for REVO NX and IOLMaster 700 (AXL: 9 *μ*m vs. 10 *μ*m; ACD: 10 *μ*m vs. 10 *μ*m; LT: 19 *μ*m vs. 20 *μ*m; CCT: 2 *μ*m vs. 2 *μ*m) [15], the presented results unequivocally support the possibility of using B-OCT measurements in calculating IOL power.

Slightly higher measurement failure rate of REVO NX (*λ* = 840 nm) as compared to SS-OCT IOLMaster 700 (*λ* = 1055 nm) in cataractous eyes is attributable to the OCT technology used in the REVO NX device rather than the B-OCT method itself. This can be better understood when illustrated with the difference between biometers based on PCI and OLCR versus SS-OCT as an analogy [16, 17]. It should be emphasized, though, that B-OCT using REVO NX has a functionality of full manual correction of identified boundaries of ocular structures, including posterior retinal boundary. Therefore, it enables a correct measurement even with very limited fundus view.  Figure 3(g) shows a successful measurement taken in an eye with a very dense posterior subcapsular cataract and almost invisible retinal pigment epithelium (RPE) boundary.

Owing to the possibility of manual RPE marker adjustment, B-OCT using REVO NX surpasses IOLMaster 700 in measuring AXL in eyes with severely disorganised macular morphology. The example shown in Figure 4 is an eye with drusenoid RPE detachment, where AXL was measured up to Bruch's membrane rather than to the elevated RPE. With IOLMaster 700, though, the posterior boundary used for AXL measurement remains unknown.  Figure 5(a) shows an eye with macula-off retinal detachment. Even in such a situation, B-OCT was capable of accurate AXL measurement, which was used for power estimation of the IOL implanted during the combined cataract extraction and vitrectomy procedure.  Figure 5(b) depicts another example of AXL measurement in a completely disorganised macular morphology due to a disciform scar.

Whereas IOLMaster 700 enables retinal imaging by allowing a low-resolution, small 1.0 mm central retinal scan, its main role is to help the clinician ensure proper fixation during measurements [18]. Thus, the device detects macular diseases with merely moderate sensitivity (between 42% and 68%) [18]. On the contrary, each B-OCT measurement using REVO NX involves high-resolution (5 *μ*m) 3 mm horizontal and vertical scans. As a result, it is possible to acquire the image of a larger retinal area with an added value of 4-fold higher resolution, which enables precise assessment of the macula. As OCT scans offering axial resolutions up to 10 *μ*m are required to enable documentation and a detailed morphologic analysis of the macula, REVO NX biometry has a potential to be a useful tool (provided that optic media are translucent enough) for the simultaneous assessment of central retina as a part of screening for macular pathology [19, 20]. If clinically indicated, a full OCT scan may be obtained immediately on the same device.

Our study has some limitations including the relatively modest number of patients with very dense cataracts, which can have an impact on the failure rate, and the small sample size of extremely short and long eyes. Also, the study does not assess the exact sensitivity and specificity of REVO NX B-OCT for detecting macular diseases before cataract surgery. The further study should then aim to better estimate the limits of the B-OCT applicability in such clinical situations.

To sum up, B-OCT showed excellent precision (intraobserver repeatability and interobserver reproducibility) for AXL, ACD, LT, and CCT measurements. The agreement between SS-OCT IOLMaster 700 biometry and B-OCT using REVO NX in AXL, ACD, LT, and CCT measurements is also outstanding. B-OCT derived values may, therefore, be used for calculating the IOL power. Whereas IOLMaster 700 is more effective than REVO NX in obtaining biometric measurements in eyes with very dense nuclear cataract and severe posterior subcapsular opacities, these differences are not caused by the limitations of B-OCT but rather by the discrepant OCT technologies used in both devices (spectral domain /*λ* = 840 nm/ vs. SS /*λ* = 1055 nm/). On the contrary, the advantage of B-OCT over IOLMaster 700 involves the possibility to manually correct the boundaries of individual structures, which enables correcting potential errors, particularly in challenging cases where IOLMaster 700 cannot guarantee the high accuracy. In addition, macular morphology scans are of significantly superior quality (over 4-fold higher resolution) and cover a larger area. Thus, with the B-OCT technology, biometry and retinal analysis can be combined in a single device. Implementing the proposed B-OCT method in commercially available OCT devices for posterior and anterior segment imaging would expand their function (all-in-one solution) enabling routine measurement of ocular axial dimensions. To the best of our knowledge, the presented method is the first spectral domain OCT-based biometry technique and the only one integrated into a standard OCT device.

## Figures and Tables

**Table 1:** Intraobserver repeatability results for B-OCT using REVO NX based on three measurements taken by 2 clinicians in a sample of 50 eyes.

Parameter	Observer	Mean ± SD	*S*_w_	TRT	CoV (%)	ICC
AXL (mm)	1st	23.93 ± 0.80	0.01	0.03	0.04	1.000
	2nd	23.93 ± 0.80	0.01	0.03	0.04	1.000

CCT (*μ*m)	1st	555.44 ± 26.56	2.01	5.58	0.36	0.994
	2nd	555.43 ± 27.08	1.75	4.85	0.32	0.996

ACD (mm)	1st	3.59 ± 0.28	0.01	0.04	0.38	0.998
	2nd	3.59 ± 0.28	0.01	0.03	0.34	0.998

LT (mm)	1st	4.02 ± 0.22	0.02	0.05	0.45	0.993
	2nd	4.02 ± 0.22	0.01	0.04	0.35	0.996

AXL: axial length; CCT: central corneal thickness; ACD: anterior chamber depth; LT: lens thickness; Sw: within-subject SD; TRT, test-retest repeatability (2.77 Sw); CoV: within-subject coefficient of variation; ICC: intraclass correlation coefficient.

**Table 2:** Interobserver reproducibility results for B-OCT using REVO NX based on the first readings from each session taken by 2 clinicians in a sample of 50 eyes.

Parameter	1st observer, mean ± SD	2nd observer, mean ± SD	*S*_w_	TRT	CoV (%)	ICC
AXL (mm)	23.93 ± 0.80	23.93 ± 0.80	0.02	0.04	0.06	1.000
CCT (*μ*m)	555.72 ± 26.55	555.54 ± 27.25	2.21	6.12	0.40	0.993
ACD (mm)	3.58 ± 0.28	3.59 ± 0.28	0.06	0.06	0.65	0.993
LT (mm)	4.02 ± 0.22	4.02 ± 0.22	0.03	0.07	0.63	0.985

AXL: axial length; CCT: central corneal thickness; ACD: anterior chamber depth; LT: lens thickness; Sw: within-subject SD; TRT, test-retest repeatability (2.77 Sw); CoV: within-subject coefficient of variation; ICC: intraclass correlation coefficient.

**Table 3:** Measurement values comparison between B-OCT REVO NX and IOLMaster 700.

	*n*	Device	Mean	SD	Paired samples *t*-test (*p* value)	Range	Difference of the means	SD for difference of the means	95% CI for difference of the means	ICC
Healthy										
Axial length (mm)	164	REVO NX	23.56	1.56	0.2664	19.11–34.48	−0.001	0.016	−0.004 to 0.001	1.000
		IOLMaster 700	23.56	1.56		19.09–34.48				
Anterior chamber depth (mm)	164	REVO NX	3.35	0.34	0.9265	1.65–4.32	0.000	0.024	−0.004 to 0.004	0.998
		IOLMaster 700	3.35	0.35		1.61–4.29				
Lens thickness (mm)	164	REVO NX	4.08	0.41	0.3398	3.30–5.34	−0.002	0.024	−0.006 to 0.002	0.998
		IOLMaster 700	4.08	0.41		3.31–5.37				
Central corneal thickness (*µ*m)	164	REVO NX	554.1	34.4	0.0552	478.1–682.3	−0.8	5.1	−1.6 to 0.0	0.989
		IOLMaster 700	553.4	35.5		477.3–684.0				

Cataract										
Axial length (mm)	111	REVO NX	23.81	1.89	0.1185	19.62–32.07	−0.003	0.022	−0.007 to 0.001	1.000
		IOLMaster 700	23.80	1.89		19.60–32.01				
Anterior chamber depth (mm)	112	REVO NX	3.10	0.41	0.3326	2.16–3.98	0.003	0.029	−0.003 to 0.008	0.997
		IOLMaster 700	3.10	0.41		2.14–4.04				
Lens thickness (mm)	111	REVO NX	4.64	0.35	0.4364	3.94–5.48	−0.002	0.025	−0.007 to 0.003	0.997
		IOLMaster 700	4.63	0.35		3.94–5.51				
Central corneal thickness (*µ*m)	115	REVO NX	546.5	33.1	<0.0001	437.8–631.0	2.7	6.4	1.5 to 3.9	0.979
		IOLMaster 700	549.2	33.6		438.2–647.0				

Difference of the means was computed by subtracting REVO NX values from IOLMaster 700 values.

## Data Availability

The data used to support the findings of this study are available from the corresponding author upon request.
